# Neuroretinitis due to *Bartonella henselae* in a returning traveler from Colombia

**DOI:** 10.1016/j.idcr.2026.e02714

**Published:** 2026-07-27

**Authors:** Elisa Milcent-Fernandez, Christian Lang, Sophie Prod’hom, Ariane Malclès, Stefano Musumeci

**Affiliations:** a Division of Tropical and Humanitarian Medicine, Geneva University Hospitals, Switzerland; b Department of Ophthalmology, Geneva University Hospitals, Geneva, Switzerland; c Faculty of Medicine, University of Geneva, Geneva, Switzerland

**Keywords:** Neuroretinitis, Cat scratch diseasease, *Bartonella hensalae*, Colombia, Cat

## Abstract

A previously healthy woman developed acute painless left-eye vision loss after travelling to Colombia, with recent febrile episodes and kitten exposure. Examination showed left neuroretinitis with papilledema and macular star. *Bartonella henselae* serology confirmed diagnosis. Treatment with doxycycline and rifampicin led to a gradual and complete visual recovery.

## Introduction

*Bartonella henselae* is a facultative intracellular pathogen. Cats are the primary reservoir, typically acquiring infection through the bite of infected cat fleas (*Ctenocephalides felis*), which mediate transmission between animals. Humans are infected through scratches, bites, or contamination of broken skin with flea-infested cat saliva. Kittens are more frequently bacteremic and play a key role in transmission. The infection classically manifests as Cat Scratch Disease, characterized by mild fever and regional lymphadenopathy. Other manifestations associated with *B. henselae* include neuroretinitis, culture-negative endocarditis, and bacillary angiomatosis, the latter occurring mainly in immunosuppressed patients [1]. Ocular involvement occurs in 5–10% of cases, while neuroretinitis accounts for 1–2% [2], [3]. Although *B. henselae* neuroretinitis has a worldwide distribution, its incidence is higher in warm and humid regions where exposure to cat fleas is more common. Unlike Cat Scratch Disease, which predominantly affects children and adolescents, neuroretinitis occurs across all age groups without a clear age or sex predilection [1], [2], [4], [5]. Neuroretinitis results from inflammatory and infectious mechanisms leading to peripapillary leakage, serous retinal detachment, and lipid exudation in a macular star pattern. Patients typically present with unilateral, painless visual loss and central or cecocentral scotoma. Optical coherence tomography commonly demonstrates peripapillary retinal thickening and subretinal fluid [2], [3], [4]. Visual recovery is usually favorable but may take months. Serologic cross-reactivity has been described with *Borrelia burgdorferi* and other pathogens, including *Coxiella burnetii*, *Chlamydia* spp., and *Brucella* spp [2]. Differential diagnoses may include also syphilis, tuberculosis, Lyme disease, sarcoidosis, and idiopathic neuroretinitis [4]. Despite its rarity, *B. henselae* remains the leading infectious cause of neuroretinitis. Evidence supporting the treatment of *B. henselae* neuroretinitis remains limited and is largely based on case reports, retrospective case series, and expert opinion. Nevertheless, doxycycline combined with rifampicin is currently the most commonly recommended regimen for neuroretinitis because of good intraocular penetration, and potential synergistic effects. Treatment of *B. henselae* depends on clinical severity: most cases are self-limited or may be treated with azithromycin for 5 days, whereas systemic forms, including neuroretinitis, require doxycycline plus rifampicin for 4–6 weeks; endocarditis necessitates prolonged doxycycline therapy combined with gentamicin during the initial 2 weeks [5], [6]. However, treatment duration should be individualized according to the patient clinical response. Evidence supporting adjunctive corticosteroid therapy remains limited, and although systemic corticosteroids have been proposed in severe cases of *B. henselae* neuroretinitis, their benefit remains controversial and they should only be considered together with appropriate antibiotic therapy [6]. Current expert recommendations advise follow-up every 2–4 weeks, including assessment of visual acuity, fundus examination, and optical coherence tomography (OCT), until resolution of optic disc edema and retinal exudates. Serological testing should not be used to monitor treatment response, as antibodies may persist for months after infection and do not correlate with clinical recovery [6].

## Case report

A previously healthy, immunocompetent woman presented with acute, painless visual loss in the left eye of one week duration after return from a one-year stay in Colombia. Three febrile episodes occurred during the preceding month. The patient reported recent adoption of a stray kitten and sustained multiple scratches and bites. She denied headache, malaise, confusion, seizures, lymphadenopathy or other constitutional symptoms. No tick bites or other animal exposure were reported. She denied dietary risk exposures but reported occasional river swimming and forest hiking. History included dengue infection in 2024. Vaccinations, including rabies immunization, were up to date. No prior ophthalmologic history was known.

**Fig. 1:**
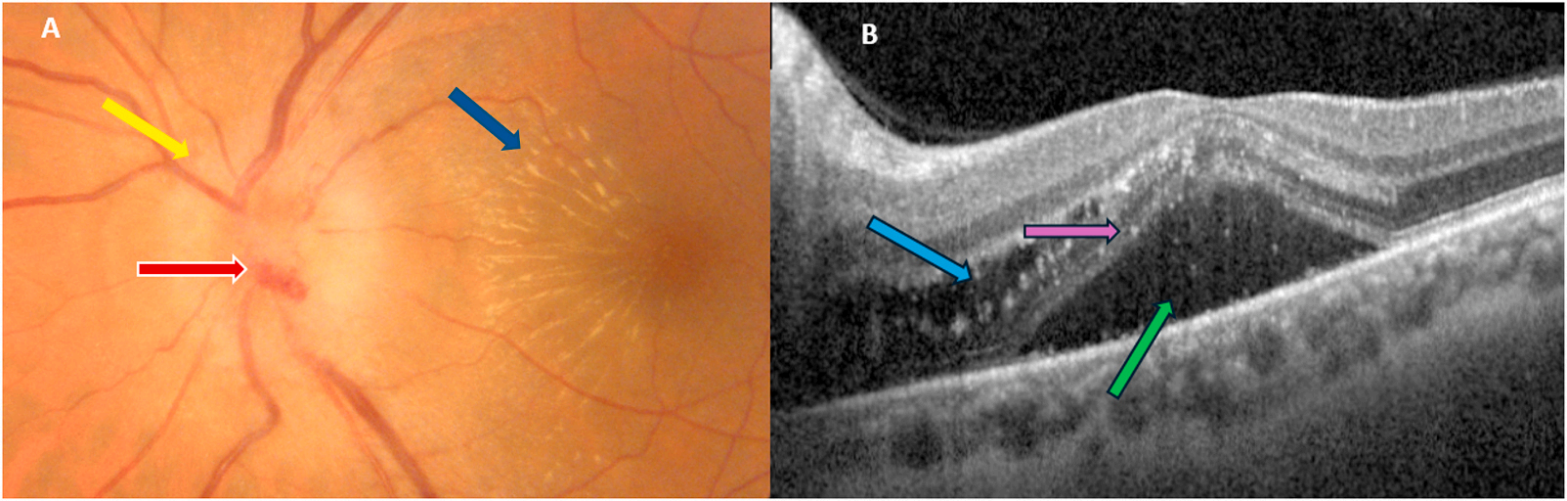
(A) Color fundus photograph of the left eye showing optic disc edema (yellow arrow), a single peripapillary hemorrhage (red arrow), and a macular star composed of hard exudates (blue arrow). (B) Macular optical coherence tomography (OCT) demonstrating intraretinal fluid (cyan arrow), subretinal fluid (green arrow), and intraretinal hyperreflective exudates (purple arrow).

Physical examination revealed a 0.5 mm scar over the dorsal aspect of the right hand, which she reported occurred three weeks before the last fever episode. Aside from mild hepatomegaly and the right-hand scratch lesion, the rest of the physical examination was unremarkable. Vitals parameters were normal, and no systemic features were present. The patient reported a left central scotoma, and best-corrected visual acuity in the left eye was reduced to 20/125. Examination of the anterior segments was unremarkable. Fundus examination revealed optic disc edema with a single peripapillary hemorrhage and macular edema associated with a partial macular star pattern **(**Fig. 1**A)**. Optical coherence tomography (OCT) confirmed macular edema, demonstrating both intraretinal and subretinal fluid accumulation, along with hyperreflective exudates **(**Fig. 1**B)**. Cerebral magnetic resonance imaging (MRI) revealed contrast enhancement of the left optic nerve head, consistent with neuroretinitis, without involvement of the remaining optic nerve segments. Given patient recent travel, an extensive diagnostic work-up was performed to exclude alternative causes of neuroretinitis. Blood investigations included complete blood count, blood cultures, malaria thick and thin blood smears, serological testing for HIV, HBV, HCV, CMV, EBV, HSV-1/2, VZV, tick-borne encephalitis virus, *Toxoplasma gondii*, *Borrelia burgdorferi*, *Bartonella henselae*, *Treponema pallidum* as well as a QuantiFERON-TB Gold assay. Cerebrospinal fluid analysis, including PCR testing for HSV-1/2, CMV, EBV, VZV, enterovirus, showed no evidence of central nervous system infection. An autoimmune antibody panel was also negative. HIV, *Treponema pallidum,* QuantiFERON-TB and *Toxoplasma gondii* testing was negative, while HBV serology was consistent with previous vaccination. Serological testing for CMV, EBV, VZV, tick-borne encephalitis virus, and *B. burgdorferi* demonstrated negative IgM and positive IgG antibodies, consistent with past infection or acquired immunity. Indirect immunofluorescence assay revealed markedly elevated IgG titers against *Bartonella henselae* (>1:2048). Based on the characteristic clinical presentation, multimodal retinal and MRI imaging, positive *B. henselae* serology, and exclusion of alternative infectious and inflammatory etiologies, a diagnosis of *B. henselae*-associated neuroretinitis was established.

Treatment with oral doxycycline and rifampicin for 4 weeks resulted in progressive and complete visual recovery.

This case underscores the importance of considering *Bartonella henselae* in returning travelers presenting with neuroretinitis and a history of cat exposure, as prompt recognition and appropriate antimicrobial therapy are associated with excellent visual outcomes.

In the present case, vitreous or aqueous humor sampling was not performed because the diagnosis was supported by the characteristic clinical presentation, multimodal retinal imaging, and positive *B. henselae* serology, without significant diagnostic uncertainty. Ocular sampling is an invasive procedure generally reserved for selected cases in which the diagnosis remains uncertain despite non-invasive investigations. Further investigations for infective endocarditis were not pursued because of the patient favorable clinical evolution and the absence of recurrent systemic symptoms during several weeks of follow-up after completion of antimicrobial therapy.

## Ethics declaration

The author(s) declare(s) that the study does not involve humans nor animal subjects.

## Consent

Patient gave oral consent.

## CRediT authorship contribution statement

**Stefano Musumeci:** Writing – review & editing, Writing – original draft, Visualization, Validation, Supervision, Formal analysis, Data curation, Conceptualization. **Ariane Malclès:** Writing – review & editing, Data curation. **Christian Lang:** Writing – review & editing, Visualization, Data curation. **Elisa Milcent-Fernandez:** Writing – original draft, Visualization, Conceptualization. **Sophie Prod’hom:** Writing – review & editing, Visualization, Validation, Data curation.

## Fundings

No specific funding was received for this work.

## Declaration of Competing Interest

The authors declare no conflicts of interest.
